# Robson Classification System Applied to Induction of Labor

**DOI:** 10.1055/s-0038-1667340

**Published:** 2018-08-02

**Authors:** Sara Vargas, Susana Rego, Nuno Clode

**Affiliations:** 1 Department of Obstetrics, Gynecology and Reproductive Medicine, Centro Hospitalar de Lisboa Norte, Lisboa, Portugal

**Keywords:** induction of labor, cesarean section, robson classification, trabalho de parto induzido, cesárea, classificação de Robson

## Abstract

**Objective** Induction of labor (IL) is a common obstetric procedure, but it is questionable whether or not it results in higher cesarean section (CS) rates. The present study aims to evaluate the impact of IL in the overall CS rates and to analyze these rates according to the method of IL employed and to the Robson group in which it was applied.

**Methods** We have conducted a retrospective study including pregnant women whose labor was induced at a tertiary hospital in 2015 and 2016. All women were classified according to the Robson Classification System (RCS). The CS rates were analyzed and compared regarding the method of IL employed.

**Results** A total of 1,166 cases were included. The CS rate after IL was 20.9%, which represented 23.1% of the total of CSs performed in 2015 and 2016. The highest CS rates were recorded in RCS groups 5 (65.2%) and 8 (32.3%). Group 2 was the highest contributor to the overall CS rate, since it represented 56.7% of the population. The intravaginal prostaglandins method was the most used (77%). Transcervical Foley catheter was the preferred method in group 5 and intravaginal prostaglandins in all the other groups. The CS rate was higher when transcervical Foley catheter was used (34.1%).

**Conclusion** Transcervical Foley catheter induction was associated with a higher rate of CS, probably because it was the preferred method used in group 5.

## Abstract (pt)

**Resumo:**

**Introdução** A indução do trabalho de parto é uma prática comum e sua associação com o aumento da taxa de cesarianas tem sido questionada. O presente estudo surge com o objetivo de avaliar o impacto da indução do trabalho de parto na taxa global de cesarianas e de analisar as taxas de cesarianas de acordo com o método de indução do trabalho de parto utilizado e com o grupo de Robson.

**Métodos** Realizamos um estudo retrospectivo com inclusão de grávidas submetidas a indução do trabalho de parto em um hospital terciário em 2015 e 2016. Todas as mulheres forram classificadas de acordo com a classificação de Robson. As taxas de cesarianas foram analisadas e comparadas em função do método de indução de trabalho de parto utilizado.

**Resultados** Foram incluídos 1.166 casos. A taxa de cesarianas após a indução do trabalho de parto foi de 20,9%, correspondendo a 23,1% do total de cesarianas realizadas em 2015 e 2016. Os grupos 5 e 8 da classificação de Robson foram os que apresentaram as maiores taxas de cesarianas (65,2% e 32,3%, respectivamente). O grupo 2 foi o que mais contribuiu para a taxa global de cesarianas, por representar 56,7% do total da população. As prostaglandinas intravaginais foram o método mais utilizado (77%). O cateter de Foley transcervical foi o método mais utilizado no grupo 5 e as prostaglandinas intravaginais nos restantes. A taxa de cesarianas foi superior quando se utilizou o cateter de Foley transcervical (34,1%).

**Conclusão** A indução do trabalho de parto com cateter de Foley transcervical associou-se a uma maior taxa de cesarianas, em provável relação com a sua maior utilização no grupo 5.

## Introduction

Induction of labor (IL) is a common obstetric procedure, and the number of cases in which labor is induced seems to have been rising in the last decades.^1^ Induction of labor should be considered whenever the risks of the pregnancy outweigh its benefits, for either the mother or the fetus.^1^ ^2^ ^3^ ^4^ Approximately 9.6% of all deliveries result from IL, but in industrialized countries this number is estimated to be ∼ 25%.^1^ The choice between multiple methods for IL, from mechanic to pharmacological, should be based on the clinical situation and the available resources. Since it is not a risk-free procedure, it should not be performed without a formal and reasoned medical indication.^5^

In the past few years, many studies have aimed to evaluate the cesarean section (CS) rates after IL. Unlike previous results, more recent studies described a reduction in CS rates after IL in term pregnancies, without an increment on perinatal morbidity.^6^ ^7^ ^8^ However, there are significant differences between the considered populations and the applied methodologies, making the results of those studies inconsistent and controversial.^4^ ^6^ ^7^ ^8^ ^9^

The progressive increase of CSs performed worldwide has become a major public health concern and multiple strategies have been discussed to reduce it.^10^ ^11^ Similarly to what happened in other countries, the Portuguese health board also adopted the Robson Classification System (RCS) in 2015.^12^ ^13^ This classification system (Table 1), initially created and proposed as a clinically relevant, reproducible and prospective instrument for evaluating, monitoring and comparing CS rates, has been recently used for other purposes.^14^ ^15^

**Table 1:** Robson classification system

Group	Description
1	Nulliparous, single cephalic, ≥37 weeks, in spontaneous labor
2	Nulliparous, single cephalic, ≥37 weeks, induced or CS before labor
3	Multiparous (excluding previous CS), single cephalic, ≥37 weeks, in spontaneous labor
4	Multiparous (excluding previous CS), single cephalic, ≥37 weeks, induced or CS before labor
5	Previous CS, single cephalic, ≥37 weeks
6	All nulliparous breeches
7	All multiparous breeches
8	All multiple pregnancies (including previous CS)
9	All abnormal lies (including previous CS)
10	All single cephalic, ≤36 weeks (including previous CS)

Abbreviations: CS, cesarean section.

In our institution, we perform around 2,300 deliveries per year, and we routinely classify all women in labor according to the RCS. In 2016, our CS rate was 24.3% and the IL rate was 26.9%. One third of all CSs performed were elective. In the present study, we have aimed to evaluate the impact of IL in the overall CS rate, as well as to analyze CS rates according to the method of IL employed and to the Robson group in which it was applied.

## Methods

The department of obstetrics and gynecology of the Hospital de Santa Maria, Centro Hospitalar de Lisboa Norte, Lisbon, Portugal, represents a tertiary university/public maternity where no CSs are performed based on maternal request.

We reviewed and analyzed the clinical data from all deliveries that occurred after IL in this institution in 2015 and 2016. Every pregnant woman was classified according to the RCS (Table 1). We have excluded all women from groups in which either no IL was performed (groups 1 and 3) or if there was a formal indication to perform an elective CS (group 9). We have also excluded all women that went into spontaneous labor and those submitted to elective CS.

The IL methods used were: 1) intravenous oxytocin (initial dose of 15 mL/hour with progressive increase until regular contractility was achieved or until reaching a maximum dose of 192 mL/hour); 2) intravaginal prostaglandins (vaginal application of 25 μg misoprostol capsules every 4 hours up to a maximum of 5 applications over 24 hours, or vaginal application of 10 mg prolonged-release pessary of dinoprostone to be withdrawn in the active stage of labor or after 24 hours); or 3) transcervical Foley catheter (Foley catheter 16 inserted after vaginal disinfection, inflated with 40 mL of distilled water, and to be withdrawn in the active stage of labor or after 24 hours, either followed or not by intravenous oxytocin or intravaginal prostaglandins).

The IL was considered a failure when no cervical changes were recorded in 48 hours. A history of previous CS represented a contraindication for IL with misoprostol and two or more CSs were a formal indication for an elective CS.

We analyzed and compared the CS rates according to each method of IL employed and each RCS group . Statistical analysis was performed using the chi-squared test on the IBM SPSS, version 22.0 (IBM Corp. Armonk, NY, USA), and *p* values < 0.05 were considered statistically significant.

## Results

Throughout the period of time considered, 1,166 pregnant patients were submitted to IL and accounted for 26.9% of all deliveries. The CS rate after IL was 20.9% (*n* = 244) and represented 23.1% of all CSs performed in 2015 and 2016.

The highest CS rates (Table 2) were recorded in groups 5 (65.2%) and 8 (32.3%). The CS rate in group 7 was 20%, and 20.6% in group 10. Group 2, which represented 56.7% of the study population, was the one that contributed the most to the overall CS rate, followed by group 5 (65.2%).

**Table 2:** Cesarean section rate after induction of labor labor according to each group of the Robson Classification System

Groups	CS rate^a^	Relative size of the group^b^	Absolute contribution to the overall CS rate^c^	Relative contribution to the overall CS rate^d^
Group 2	22.4 (148/661)	56.7 (661/1,166)	12.7 (148/1,166)	60.7 (148/244)
Group 4	8.3 (27/326)	28 (326/1,166)	2.3 (27/1,166)	11.1 (27/244)
Group 5	65.2 (43/66)	5.7 (66/1,166)	3.7 (43/1,166)	17.6 (43/244)
Group 6	0 (0/4)	0.3 (4/1,166)	0 (0/1,166)	0 (0/244)
Group 7	20 (1/5)	0.4 (5/1,166)	0.1 (1/1,166)	0.4 (1/244)
Group 8	32.3 (10/31)	2.7 (31/1,166)	0.9 (10/1,166)	4.1 (10/244)
Group 10	20.6 (15/73)	6.3 (73/1,166)	1.3 (15/1,166)	6.2 (15/244)
Total	–	100 (1,166/1,166)	20.9 (244/1,166)	100 (244/244)

Abbreviations: CS, cesarean section; IL, induction of labor.

a % (number of CS in the group/number of women in the group);

b % (number of women in the group/total number of IL);

c % (number of CS in the group/ total number of IL);

d % (number of CS in the group /total number of CS).

The intravaginal prostaglandins method was the most used for IL (77%), followed by transcervical Foley catheter (15.9%) and intravenous oxytocin (7.1%) (Table 3). When compared with other methods, the transcervical Foley catheter was associated with a higher CS rate (*p* = 0.042 and *p *< 0.001). There was no significant difference between using oxytocin and intravaginal prostaglandins (*p* = 0.427). The transcervical Foley catheter was the preferred method in group 5 (74.2%), while the intravaginal prostaglandins method was the most used in all other groups (79.6–100%). The CS rate after IL with intravaginal prostaglandins was never > 25.9%.

**Table 3:** Methods for induction of labor and cesarean section rates according to each group of the Robson Classification System

Groups	Transcervical Foley catheter		Intravenous oxytocin		Intravaginal prostaglandins	
	IL^a^	CS rate^b^	IL^a^	CS rate^b^	IL^a^	CS rate^b^
Group 2	15.6 (103/661)	22.3 (23/103)	4.8 (32/661)	6.3 (2/32)	79.6 (526/661)	23.4 (123/526)
Group 4	7.1 (23/326)	21.7 (5/23)	8.6 (28/326)	7.1 (2/28)	84.4 (275/326)	7.3 (20/275)
Group 5	74.2 (49/66)	67.4 (33/49)	22.7 (15/66)	66.7 (10/15)	3 (2/66)	0 (0/2)
Group 6	0 (0/4)	0 (0/0)	0 (0/4)	0 (0/0)	100 (4/4)	0 (0/4)
Group 7	0 (0/5)	0 (0/0)	0 (0/5)	0 (0/0)	100 (5/5)	20 (1/5)
Group 8	3.2 (1/31)	100 (1/1)	9.7 (3/31)	66.7 (2/3)	87.1 (27/31)	25.9 (7/27)
Group 10	12.3 (9/73)	11.1 (1/9)	6.9 (5/73)	40 (2/5)	80.8 (59/73)	20.3 (12/59)
Total	15.9 (185/1,166)	34.1 (63/185)	7.1 (83/1,166)	21.7 (18/83)	77 (898/1166)	18.2 (163/898)

Abbreviations: CS, cesarean section; IL, induction of labor.

a % (number of IL in the group with each method/total number of women in the group);

b % (number of CS in the group with each method/number of IL in the group with each method).

## Discussion

Throughout the period of time considered, the IL rate (26.9%) was similar to what has been reported by other countries.^1^ The observed CS rate after IL (20.9%) was lower than the overall CS rate of our institution and may be explained by the fact that we are a tertiary hospital, capable of monitoring high-risk pregnancies that often have a formal indication for an elective CS. The exclusion of elective CSs from our sample may justify, in part, the lower value of the CS rate observed after the IL. Furthermore, it could also be related to the meticulous case choice for IL, based on the conviction of a high probability of a vaginal delivery.

The intravaginal prostaglandins method was the most used method for IL in all groups, except group 5, in which misoprostol cannot be used. Intravaginal prostaglandins can be used in different doses, formulations and routes of administration. Despite being off-label and associated with uterine hyperstimulation, its use according to our protocol is one of the most effective for IL.^16^ When compared with other methods, the IL with misoprostol results in higher rates of vaginal deliveries and in lower rates of CS, especially when the Bishop score is not favorable (< 6).^1^ Therefore, it is actually the preferred method for IL.

The transcervical Foley catheter was the second most used method for IL (15.9%) and the one associated with a higher CS rate (34.1%). Since it is linked to a lower risk of uterine hyperstimulation and it is not contraindicated for IL in women with a previous CS, it was the preferred method in group 5 (74.2%). In the past, transcervical Foley catheter was replaced by pharmacological agents, but recently it has showed promising results, either alone or in combination with other methods, especially when used with unfavorable Bishop scores.^17^ ^18^ ^19^

Oxytocin is a commonly used method for IL worldwide, either alone or in combination with other methods. However, when used alone, it is less effective than intravaginal prostaglandins.^20^ In our institution, we use it in a residual subgroup of women with favorable Bishop scores. Furthermore, it is also an option for IL in women with a previous CS, which might explain the CS rate of 21.7%. According to the World Health Organization (WHO), the use of oxytocin alone for the IL should be reserved for situations when intravaginal prostaglandins are not available.^1^

As expected, groups 2 and 4 represented 84.7% of our sample. Since group 2 (*n* = 661) accounted for 56.7% of the entire population, and even with its CS rate of 22.4%, it was the biggest contributor (60.7%) to the overall CS rate. After comparing these results with the CS rate of 10% recorded in group 1 in our institution since 2014, IL seems to be associated with a higher CS rate. Nevertheless, and according to Robson,^21^ this number continues to be lower than the expected CS rate of 25 to 30% after IL in this group.

Much like what has been reported by other studies, group 5 revealed the highest CS rate, even higher than expected.^18^ ^19^ ^21^ ^22^ These results are connected to the fear of uterine rupture and to the difficulty of choosing an IL method that is not contraindicated and, therefore, that is also less effective.^23^ ^24^ Despite the small size of this group (*n* = 66), it was the second biggest contributor (17.6%) to the overall CS rate. With the progressive increase of CSs performed worldwide, we also expect in the future a higher number of women and of IL in this group.^25^ ^26^ In this setting, the use of the transcervical Foley catheter is more effective than the use of oxytocin alone. However, the heterogeneity of the characteristics of these women, both clinical and obstetric, interferes with and adds difficulty to the choice of an ideal method for IL in group 5.

Group 10 (*n* = 73) was the third largest group (6.3%) of our sample, which might be related to the characteristics of our neonatal intensive care unit. Despite the fact that the CS rate tends to be higher with lower gestational ages, the CS rate in this group (20.6%) was acceptable.^17^ ^27^

Group 4 (*n* = 326) represented 28% of our sample and had a CS rate of 8.3%, higher than the expected 4% to 6%.^21^ The comorbidities of this population might have contributed to this number.

Since we represent a national reference in the field of medically assisted procreation, the IL rate of twin pregnancies is significant (*n* = 31). Thus, we also have practice in twin vaginal deliveries. Despite the high CS rate recorded in group 8 (32.3%), it is still lower than what has been recorded in previous studies.^21^ ^28^ Because there is a lack of studies regarding the best method for IL in this group, intravaginal prostaglandins were the most used (87.1%).

In the past few years, the number of cephalic versions and breech vaginal deliveries performed in our institution has been increasing with encouraging outcomes, which contributed to the progressive reduction of CS rates in groups 6 and 7.^29^ ^30^ Nevertheless, the numbers of ILs in these groups are residual, due to the reduction of training and to the careful selection of cases. However, we have noted a low rate of CSs in these groups (0% and 20%, respectively).

The retrospective design of our study represents a limitation. Additionally, we did not evaluate all the demographic and clinical characteristics of our sample, which makes it a challenge to compare our results to those reported by previous studies. While we acknowledge the possible difference in effectiveness between using the transcervical Foley catheter alone or in combination with other methods, we have decided to analyze this data together, due to the small number of cases.

The RCS allowed for a better comparison between different methods of IL, especially with regard to their choice in each group. The differences between the CS rates in each group, as well as a comparison to those reported by previous studies, allow us to identify target groups for a better approach when IL is considered (group 5).

Although the CS rate after IL was lower than the overall CS rate of our institution in the considered period of time, we cannot claim that IL is associated with a reduction in CS rates, given the high number of elective CSs performed.

## Conclusion

The number of CSs performed after IL corresponded to 23.1% of the total. The intravaginal prostaglandins method was the most used and also the most effective. The transcervical Foley catheter was the method associated with a higher CS rate, probably because it was the preferred method in group 5. The RCS seems to be useful in this evaluation, as it simplifies the stratification of the population and the interpretation and comparison of the results.
